# The Role of Ultrasonography in the Process of Weaning from Mechanical Ventilation in Critically Ill Patients

**DOI:** 10.3390/diagnostics14040398

**Published:** 2024-02-12

**Authors:** Lou’i Al-Husinat, Basil Jouryyeh, Ahlam Rawashdeh, Chiara Robba, Pedro Leme Silva, Patricia Rieken Macedo Rocco, Denise Battaglini

**Affiliations:** 1 Department of Clinical Medical Sciences, Faculty of Medicine, Yarmouk University, Irbid 21163, Jordan; loui.husinat@yu.edu.jo; 2 Faculty of Medicine, Yarmouk University, Irbid 21163, Jordan; bassiljoureyah978@gmail.com (B.J.); ahlamrawashdeh00@gmail.com (A.R.); 3 Anesthesia and Intensive Care, IRCCS Ospedale Policlinico San Martino, 16132 Genova, Italy; kiarobba@gmail.com; 4 Department of Surgical Sciences and Integrated Diagnostics (DISC), University of Genova, 16132 Genova, Italy; 5 Laboratory of Pulmonary Investigation, Carlos Chagas Filho Biophysics Institute, Federal University of Rio de Janeiro, Rio de Janeiro 21941, Brazil; pedroleme@biof.ufrj.br (P.L.S.); prmrocco@gmail.com (P.R.M.R.)

**Keywords:** weaning, mechanical ventilation, lung ultrasound, diaphragm ultrasound, laryngeal ultrasound, echocardiography, physiotherapy

## Abstract

Weaning patients from mechanical ventilation (MV) is a complex process that may result in either success or failure. The use of ultrasound at the bedside to assess organs may help to identify the underlying mechanisms that could lead to weaning failure and enable proactive measures to minimize extubation failure. Moreover, ultrasound could be used to accurately identify pulmonary diseases, which may be responsive to respiratory physiotherapy, as well as monitor the effectiveness of physiotherapists’ interventions. This article provides a comprehensive review of the role of ultrasonography during the weaning process in critically ill patients.

## 1. Introduction

Weaning from mechanical ventilation (MV) is the gradual process of transitioning a patient from dependence on a ventilator to breathing independently, with the ultimate goal of successful liberation from the ventilator. This process consists of various stages, including readiness testing, weaning proper, and extubation [1]. Successful weaning and extubation is generally defined as the absence of need for reintubation for at least 48 h after removal of the endotracheal tube [2,3], or, as defined by the WIND study, when the patient remains extubated without reintubation or dies 7 days after extubation [4]. Nevertheless, outcomes can vary depending on the patient’s readiness and underlying medical condition, with only 65% being successfully weaned, according to the most recent literature [1]. The success or failure of weaning attempts can be influenced by various underlying pathophysiological factors [5]. Critically ill patients often present with a combination of clinical conditions, including lung and cardiovascular issues, chest wall changes, peripheral muscle weakness, reduced respiratory drive, and neurological impairments that affect the transition from MV to spontaneous breathing [6]. 

Defining the best time for extubating critically ill patients poses some challenges because early extubation increases the incidence of weaning failure, necessitating reintubation, which exposes patients to hemodynamic instability and respiratory distress, which should be avoided [7]. However, delaying extubation increases the duration of MV and carries other risks, such as tracheal damage, ventilator-associated pneumonia, and barotrauma [8]. Therefore, ensuring patients wean safely and effectively is critical for patient outcomes.

Current guidelines suggest several bedside indices to predict successful weaning and extubation. However, none of these indexes has been proven to be optimal in predicting weaning success [2]. Implementing a spontaneous breathing trial (SBT) to predict the outcomes of weaning is also recommended [9]. SBT is defined as a period of observation during which patients breathe through a T-piece with adequate supplemental oxygen or with reduced pressure support (PS), whether or not supplemented by 5 cm H_2_O positive end-expiratory pressure (PEEP), in individuals with a normal body mass index (BMI) [3,10]. Notably, 20% of the highest-risk patients who undergo a successful SBT still require a subsequent re-intubation [10]. Reintubation rates did not differ where a SBT was carried out via a T-piece or pressure support ventilation (PSV) in high-risk patients [11]. Moreover, while SBT is generally considered safe and can reduce the duration of MV with its associated complications and intensive care costs [12], it may not necessarily contribute to a deeper understanding of the root causes behind weaning failure. This is where ultrasonography becomes useful, as it allows for direct bedside evaluation of many of these causes, such as heart, lung, or muscle dysfunction, by providing real-time, non-invasive imaging of different body organs. It utilizes high-frequency sound waves that differ according to the selected probe to visualize its internal structures and monitor its dynamic changes [13].

The aim of this review is to discuss the multiple uses of ultrasonography (US) in assessing the process of weaning from MV. We will focus on its role in evaluating lung aeration, respiratory muscle function, airway readiness, hemodynamic stability, and the effectiveness of respiratory physiotherapy.

## 2. Ultrasonographic Assessment during the Weaning Process

**Figure 1:**
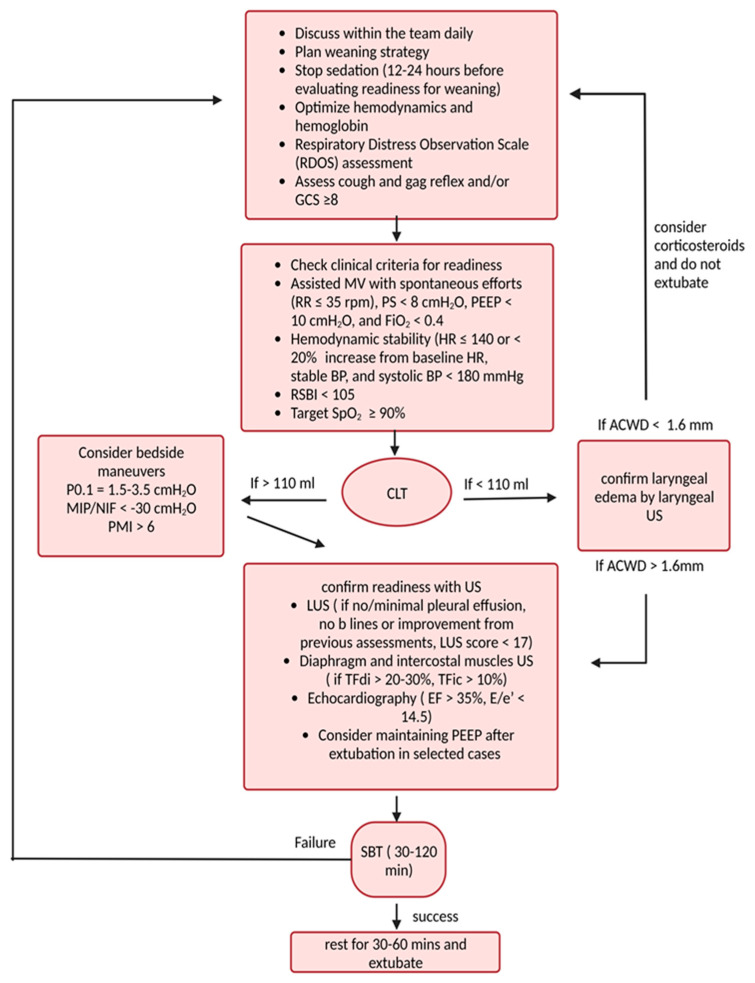
How to integrate ultrasound (US) into the process of weaning from mechanical ventilation. ACWD = air column width difference, BP = blood pressure, CLT = cuff leak test, EF = ejection fraction, E/e’ = left ventricular filling pressure, FiO_2_ = fraction of inspired oxygen, GCS = Glasgow coma scale, HR = heart rate, LUS = lung ultrasound, MIP = maximal inspiratory pressure, MV = mechanical ventilation, NIF = negative inspiratory force, PEEP = positive end-expiratory pressure, PMI = pressure muscle index, PS = pressure support, P0.1 = airway occlusion pressure to detect inspiratory effort at the bedside, RDOS = respiratory distress observation scale, RR = respiratory rate, RSBI = rapid shallow breathing index, SBT = spontaneous breathing trial, SpO_2_ = peripheral saturation of oxygen, TFdi = diaphragmatic thickening fraction, TFic = intercostal muscle thickening fraction.

By enabling clinicians to directly observe various organ functions at the bedside, ultrasonography can enhance the accuracy of weaning assessments, guide clinical decision-making, and contribute to more timely and successful liberation from MV [3]. To evaluate these functions, intensivists need to possess basic and advanced ultrasound skills to effectively assess and wean off patients from mechanical ventilation [14]. See Figure 1 for the main ultrasonographic indices of weaning failure, the timing, and how to use US during the weaning process.

### 2.1. Ultrasonographic Assessment of Lung Aeration 

Lung ultrasound (LUS) has emerged as a promising tool to aid in the process of weaning. It provides an immediate understanding of both lung aeration and ventilation conditions at the bedside without needing to transfer patients or expose them to any radiation. LUS is especially appropriate to identify potential factors related to changes in lung aeration that may contribute to weaning failure [15]. 

LUS relies on the air-to-fluid ratio within the lung parenchyma to provide informative images. In regions where air is entirely absent (i.e., consolidation), LUS successfully gives an accurate image of the lung tissue, possibly indicating the presence of disease. Conversely, when air is present in the tissue, various image artifacts can arise. Depending on the artifact produced, it can either represent lung tissue with normal aeration or reduced relative aeration, irrespective of the specific underlying pathological condition. Reduced relative aeration can occur due to factors like air loss (leading to atelectasis) or the accumulation of fluid within the interstitial or alveolar spaces [16].

In a typical LUS assessment, twelve thoracic regions are examined, six for each side of the chest. These regions are delineated using the anterior and posterior axillary lines as references, and subsequently, each zone is subdivided into upper and lower segments. The ultrasound probe is then passed along each intercostal space within these twelve regions, providing a standardized method for imaging the entire thorax [17,18]. 

The results of the LUS assessment can be classified into four identifiable patterns, each representing varying degrees of lung aeration. To semi-quantify these variations, multiple scoring systems have been proposed. In the intensive care unit (ICU), the most employed system is the “Bedside Lung Ultrasound in Emergency” (BLUE) protocol, which assigns a specific score (from 0 to 3) to each stage, as detailed in Table 1 [3,19,20].

The total LUS score is the sum of the scores from the twelve thoracic regions, with the lowest possible score of 0 (indicating normal aeration in all regions) and the highest possible score of 36 (suggesting consolidation in all regions) [19]. Bouhemad et al. originally introduced the LUS score to assess patterns of lung aeration. Subsequently, it has also been used to predict the success of weaning patients from MV [8].

#### 2.1.1. Application of LUS to Predict the Outcome of Weaning from Mechanical Ventilation

As previously discussed, the decision regarding the optimal time to initiate the weaning process remains a dilemma for most mechanically ventilated patients. Hence, recent research has focused on the potential role of LUS in evaluating patient readiness and predicting weaning outcomes. A prospective study by Soummer et al. aimed to predict post-extubation distress following discontinuation of MV, which encompasses the need for reintubation or rescue noninvasive ventilation (NIV) within 48 h. The researchers examined 100 patients and utilized LUS to evaluate lung aeration before and after a 60-min SBT, in addition to 4 h post-extubation. The LUS score was used to quantify lung aeration. Significant lung derecruitment occurred during a one-h SBT, and this was more pronounced in patients who developed post-extubation distress. Moreover, among patients successfully passing the SBT, a LUS score of ≤12 at the end of the SBT strongly indicated a higher likelihood of post-extubation success. Conversely, a LUS score of ≥17 at the end of the SBT was highly predictive of post-extubation distress [20].

In a complementary study conducted by Shoaeir et al., the potential of LUS in predicting weaning outcomes was examined in 50 intubated patients aged 18 years and older who had been intubated for at least 48 h and met the criteria for SBT. Lung aeration scoring using LUS was performed before, during the SBT, and after extubation. The patients were closely monitored for 48 h post-extubation to assess the outcome and subsequently divided into two distinct groups: the weaning failure group and the weaning success group. Patients in the weaning success group exhibited significantly lower baseline LUS aeration scores when compared to those in the weaning failure group. Additionally, important changes in aeration scores were observed during the SBT in the weaning failure group. Ultimately, the results demonstrated that a LUS aeration score shows promising potential in forecasting weaning failure in critically ill patients, as scores ≥ 18 had a good predictive value for weaning failure, while a LUS aeration score ≤ 11 had a good prediction for weaning success. Scores between these values were inconclusive in predicting weaning outcomes [21].

A comprehensive systematic review and meta-analysis conducted by Llamas-Álvarez et al. in 2017, which involved a cohort of 1071 patients admitted to ICUs and subjected to MV for at least 24 h, reported that the use of lung and diaphragmatic ultrasound can aid in predicting the outcomes of weaning. However, the limited number of studies may lead to a degree of uncertainty when interpreting these findings [22]. A recent observational study by Kundu et al. also underlined the promising ability of LUS to anticipate the outcomes of the weaning process. The study demonstrated that employing an inclusive ultrasound protocol incorporating lung, diaphragm, and cardiac sonography reliably predicted the likelihood of weaning failure [23].

As a suggestion, we advocate for the routine integration of LUS in clinical protocols for ventilatory management. Given its effectiveness in providing real-time insights into lung function during both controlled and assisted ventilation, as well as during SBT, LUS stands as a valuable tool. Specifically, its application before extubation proves crucial in informing decisions such as whether to implement positive end expiratory pressure (PEEP) after extubation. This non-invasive approach offers clinicians a practical means of tailoring interventions and optimizing respiratory care in critical settings. However, it is essential to emphasize the need for rigorous clinical trials to affirm the efficacy and reliability of LUS in these contexts, ensuring evidence-based integration into standard clinical practice.

### 2.2. Ultrasonographic Assessment of Diaphragmatic Function

The diaphragm has a significant role as a primary inspiratory muscle involved in breathing. This muscle accounts for about 75% of the ventilation process at rest. However, critical conditions such as hypotension, insufficient oxygen levels, systemic infections, and the requirement of MV may impair diaphragm function [24]. 

MV can cause diaphragmatic atrophy and dysfunction. This condition, referred to as ventilator-induced diaphragmatic dysfunction (VIDD), may affect the ability of the patient to discontinue MV [25]. VIDD may occur shortly after the initiation of ventilation and worsen over time, depending on the mode of ventilation used and other associated risk factors [26]. As a result, it is important to assess diaphragmatic function to predict the patient’s ability to wean from MV and sustain spontaneous breathing.

Ultrasound has been implemented as a non-invasive bedside tool to visualize the diaphragm for at least four decades. Nevertheless, only recently has ultrasound been used to assess the size and function of the diaphragm when the patient is under MV [27,28]. Ultrasound imaging has two main modes: brightness mode (B-mode) and motion mode (M-mode). B-mode provides detailed two-dimensional images of structures for anatomical assessment, while M-mode focuses on capturing the movement of structures over time using a dynamic graph and can accurately measure the diaphragmatic displacement over the respiratory cycle. Applying these modes to visualize the diaphragm allows assessment of diaphragmatic thickness (Tdi), primarily using the B-mode, and diaphragmatic excursion (E), mainly using the M-mode [29,30]. When examining critically ill patients, it is typically sufficient to assess only the right diaphragm unless there is suspicion of dysfunction on the left side, in which case both sides should be assessed [31]. It is generally easier to obtain a clear view of the right side due to the liver providing an excellent ultrasound window compared to the left side due to the poor acoustic window of the spleen [29,32].

#### 2.2.1. Diaphragmatic Thickness (Tdi) and the Thickening Fraction (TFdi)

To assess diaphragmatic atrophy and contraction, it is essential to assess diaphragmatic thickness (Tdi) and diaphragmatic thickening fraction (TFdi), respectively [33,34].

Tdi is measured using a high-frequency linear probe (≥10 MHz) placed on the zone of apposition (ZA) where a patient’s diaphragm meets the thoracic cage. The probe should be angled perpendicular to the lateral chest wall and placed between the midaxillary and anterior axillary lines at the eighth or ninth intercostal space [29,35]. The estimated distance between the skin and the diaphragm in this area ranges from 0.8 cm to 4.9 cm. Individuals with a higher body mass index exhibit an increase in the depth of the diaphragm [31]. The reference values for Tdi in critically ill patients were reported in various studies. Goligher et al. found that the mean Tdi was 2.4 ± 0.8 mm [36], whereas Schepens et al. reported it was slightly lower, 1.9 ± 0.4 mm [37].

The diaphragm is identified as a hypoechoic structure surrounded by two hyperechoic lines: the peritoneum and pleura membranes. These lines representing the outer layers are excluded when measuring thickness as they serve as borders. The fibrous layer located in the center of the diaphragm is often identified by a third hyperechoic line that can be observed within the non-echogenic layer [35]. Tdi is measured at the end of expiration (Tdi-exp) and inspiration (Tdi-insp) as the distance between the diaphragmatic pleura and the peritoneum using the B-mode or M-mode [26].

The diaphragmatic function is closely linked to inspiratory thickening. As a consequence, for better detection of diaphragmatic dysfunction (DD), it is essential to measure diaphragmatic thickness both at the end of inspiration (Tdi-insp) and at the end of expiration (Tdi-exp). This measurement is referred to as the thickening ratio (TR), i.e., thickness at end inspiration divided by thickness at end expiration. Additionally, some researchers used the concept of thickening fraction (TFdi), calculated using the formula below multiplied by 100 [35]:

$$\mathrm{TFdi}=\frac{\left(\mathrm{Tdi}-\mathrm{insp}\right)-(\mathrm{Tdi}-\exp)}{\left(\mathrm{Tdi}-\exp\right)}$$

#### 2.2.2. Diaphragmatic Excursion (E) 

Diaphragmatic excursion (E) is measured using a low-frequency convex or phased array probe (1–5 MHz) positioned on the front subcostal area between the mid-clavicular line and the anterior axillary line. The lower frequency provides enhanced depth, albeit at the cost of reduced spatial resolution [29,38]. The assessment of the left and right hemidiaphragms can be carried out using the spleen and liver windows, respectively. To assess the right hemi-diaphragmatic excursion, the probe should be angled medially, cranially, and dorsally so that the ultrasound beam reaches the posterior third of the diaphragm, showing the right hemidiaphragm as a thick, curved, hyperechoic line in B-mode. Then, the M-mode exploration line must be placed perpendicular to the diaphragmatic dome in order to obtain maximum excursion. The transducer must be held in place, and the patient is instructed to perform quiet breathing (QB), deep breathing (DB), and voluntary sniffing (VS). The measurement of diaphragmatic excursion amplitude involves positioning calipers at the lower and upper points of the inspiratory slope of the diaphragm [38]. Movement of the liver and spleen can be used as an alternative when visualizing the diaphragm is difficult from the subcostal window. For this reason, an intercostal window is recommended at the zone of apposition in B- or M-mode, using a low-frequency probe [39,40]. Since there can be differences in how the diaphragm and the area below it move together [41,42], this approach is better for describing how the diaphragm moves rather than trying to measure it precisely [32].

Measuring excursion can only be carried out in spontaneously breathing patients, as during assisted ventilation, passive displacement cannot be distinguished from active displacement due to driving pressures [29]. Excursion positively correlates with lung inspiratory volumes; thus, its values increase during forced inspiratory breathing [28]. Furthermore, in healthy volunteers, diaphragmatic excursion varies with height, weight, sex, and age and can be reliably measured in a recumbent or supine position [43]. In a study published in 2022, Kabil et al. defined the normal range of diaphragmatic excursion for the normal population in 757 healthy volunteers. They found out that the mean hemi-diaphragmatic excursion on the right side was 5.54 ± 1.26, 2.90 ± 0.63, and 2.32 ± 0.54 cm for deep breathing, sniffing, and quiet breathing, respectively, while the mean hemi-diaphragmatic excursion on the left side was 5.30 ± 1.21, 2.97 ± 0.56, and 2.35 ± 0.54 cm for deep breathing, sniffing, and quiet breathing, respectively [43]. 

During inspiration, the diaphragm moves downwards towards the probe. If there is absent or reduced movement that falls below normal reference values or movement that goes against the probe, it indicates dysfunction of the diaphragm [26]. The success rate for visualizing E during tidal breathing (>95%) is high, while during maximal breathing visualization is more difficult, especially on the left side [44].

Lastly, we found variation in using diaphragmatic measurements (TFdi, E) in defining diaphragm dysfunction, as detailed in Table 2.

#### 2.2.3. Application of Diaphragmatic Ultrasound in Weaning from Mechanical Ventilation

During the weaning process, clinicians use objective clinical criteria and sometimes physiological tests like diaphragmatic ultrasonography to predict whether a patient is likely to tolerate weaning in a process called readiness testing. When considering the predictive role of diaphragmatic ultrasonography in assessing the preparedness for weaning from MV, findings are conflicting [26]. Numerous studies suggested that diaphragmatic excursion cut-off values of 10 mm to 13 mm or more [49,50,51,52] and diaphragmatic thickening fraction values of 20 to 30% or more are predictable for successful extubation [52,53,54]. However, considerable heterogeneity across the studies regarding the definitions of weaning or extubation failure, patient position during ultrasonography, type of SBT, and the type of measure used to assess the outcome (E, TFdi, or both) is found, thus creating challenges in forming definitive conclusions about the effectiveness of diaphragmatic ultrasound for predicting weaning success. In recent years, multiple systematic reviews and meta-analyses were conducted to establish evidence on the usefulness of diaphragmatic ultrasound in predicting the success or failure of weaning in patients undergoing MV, and their results suggested adequate accuracy of diaphragmatic ultrasound in predicting weaning success [55,56,57]. In this line, in 2023, Parada Gereda et al. included 19 studies in a meta-analysis of 1204 patients. The sensitivity and specificity of diaphragmatic excursion to predict weaning success were both 80%, and the area under the summary receiver operating characteristic (ROC) curve was 0.87. For the TFdi, sensitivity and specificity were 85% and 75%, respectively, and the area under the ROC curve was 0.87. However, these studies showed a great heterogeneity, suggesting that further studies are needed to better evaluate the role of diaphragm ultrasound as a predictor of weaning from MV [58].

Assisted mechanical ventilation (AMV), exemplified by pressure support ventilation (PSV), is commonly applied to critically ill patients to relieve the respiratory muscles and prevent muscle atrophy [59]. The patient’s inspiratory muscles generate a variable amount of work, with the ventilator supplying the remainder [60]. Under-assistance may result in fatigue and discomfort, while over-assistance can lead to patient-ventilator asynchrony [61] and ventilator-induced diaphragm dysfunction [62]. Measuring the patient’s effort during assisted breathing is challenging in clinical settings, and direct clinical assessment of the diaphragm is not feasible. This is when researchers began considering the use of diaphragmatic ultrasonography for this purpose. Umbrello et al. conducted a pilot study aimed at evaluating the performance of TFdi and E to assess the relative contribution of patients’ effort during AMV. They concluded that, in patients undergoing AMV, TFdi proved to be an effective indicator of alterations in inspiratory muscle effort in response to adjustments in the PS level, in contrast to E, which should not be used for the quantitative assessment of diaphragmatic contractile activity. Additional investigations are needed to determine if this remains valid in a larger patient population encompassing various diseases [63].

In summary, we suggest including diaphragm ultrasound in MV weaning protocols. This can be especially beneficial during the shift from controlled to assisted ventilation, achieving smoother transitions in mechanical ventilation to optimize patient response in the weaning process. Rigorous investigations will contribute to a more comprehensive understanding of the potential benefits and applications of diaphragm ultrasound in clinical practice.

#### 2.2.4. Tissue Doppler Imaging 

Tissue Doppler imaging (TDI) is an ultrasonographic technique used to detect alterations in the frequency of ultrasound signals reflected by mobile structures. While it has been extensively employed for evaluating cardiac performance, its application for diaphragm assessment in both adults and neonates has been a more recent development [64,65]. Recently, Soilemezi et al. and Cammarota et al. demonstrated that TDI-derived parameters effectively distinguished between patients who successfully completed the weaning trial and those who did not [66,67]. However, to confirm these results, further clinical trials should be conducted to address the role of TDI in predicting extubation failure.

#### 2.2.5. Speckle Tracking Ultrasound

Speckle Tracking Ultrasound (STUS), also known as strain imaging, involves tracking ultrasound speckles over time. These speckles represent regions of muscle tissue with relatively stable gray patterns in ultrasound imaging. STUS software works by tracking a group of speckles throughout the contractile cycle and measuring their displacement and deformation in relation to each other. The extent of deformation is referred to as ‘strain,’ while “strain rate” quantifies the deformation velocity [26,32]. A recent study by Oppersma et al. has shown a strong correlation between strain, strain rate, and trans-diaphragmatic pressure in healthy individuals [68]. STUS offers a significant advantage over TDI because it remains unaffected by the angle between the ultrasound beam and the tissue motion direction. While STUS applicability has been investigated for assessing diaphragm movement in various scenarios, further research is warranted, particularly in individuals with abnormal diaphragmatic function or critical illness. Comparisons with electromyography or trans-diaphragmatic pressure measurements may also be necessary to validate its applicability [26,32].

#### 2.2.6. Shear Wave Elastography 

Shear wave elastography (SWE) is an innovative technique that has recently been employed for diaphragm evaluation. This technique involves inducing tissue deformation through the generation of a focused acoustic impulse beam by the ultrasound probe. As a result, a measurable shear wave is produced, which can be converted into a shear modulus (SM). The higher the SM, the greater the tissue stiffness [26]. Utilization of SWE for assessing the diaphragm could hold clinical significance, as variations in muscle stiffness may mirror changes in muscle physiology, such as injury or fibrosis [32]. In a recent study on healthy subjects, Bachasson et al. found that the mean trans-diaphragmatic pressure (Pdi) correlated with the mean SM and that any change in diaphragmatic stiffness evaluated by SWE reflected changes in Pdi [69]. In a more recent study by Fossé et al., researchers confirmed the conclusion of Bachasson et al. that there is a correlation between Pdi and SM of the diaphragm, demonstrating that SWE has the potential to substitute Pdi in patients who are on mechanical ventilators [12]. Therefore, SWE may be a new technique to gauge diaphragmatic effort. Moreover, SWE is a more precise and consistent method compared to the assessment of echogenicity, which can be greatly influenced by ultrasound settings such as gain and contrast adjustments [32].

### 2.3. Ultrasonographic Assessment of Parasternal Intercostal Muscles

Patients who have weakness of the diaphragm may compensate for diaphragmatic dysfunction with parasternal muscle activity. The use of ultrasound to measure parasternal intercostal muscle thickening (Tic) and intercostal muscle thickening fraction (TFic), and the combination of these measurements with diaphragmatic ultrasound, has been used to predict the ability of patients to wean from MV [70]. 

The parasternal intercostal muscles can be assessed by a linear probe (10–15 MHz), positioned at a lateral distance of 3–5 cm from the sternum. The probe should be oriented transversally in the sagittal plane, precisely between the 2nd and 3rd ribs. For example, in a patient positioned supine with a 20° head-up angle, the linear probe is placed at a right angle to the front surface of the thorax in the longitudinal scan [70,71]. Ultrasound assessment of the parasternal intercostal muscles is performed mostly on the right side, as it is more feasible [72]. Similar to diaphragmatic ultrasound, the use of M-mode and B-mode ultrasonographic techniques can be applied to evaluate intercostal muscles.

#### 2.3.1. B-Mode Ultrasonography

Using the B mode, the pleural line is easily visualized, forming a component of the recognizable “bat sign” [72]. Slightly above the pleural line, the parasternal intercostal muscle can be recognized as a biconcave structure with three layers. These layers consist of two linear hyperechoic membranes extending from the anterior and posterior aspects of the adjacent ribs, along with a central portion displaying muscle echotexture [70,73]. With this mode, intercostal muscle thickness (Tic) can be measured between the inner and outermost hyperechogenic layers of the muscle fascial borders. However, thickness measurements should be performed at the craniocaudal midpoint between the ribs, where the difference in the curvature between the two surfaces is the least [74].

Furthermore, using the grayscale, ultrasound indirectly gives qualitative information on muscle composition [75]. Additionally, higher muscle echogenicity indicates poorer muscle quality [73]. However, when compared to other measures, echogenicity measurements are more influenced by observer-dependent factors, and so, a rigorous ultrasound setting must be selected before obtaining the image to standardize the methods [74].

#### 2.3.2. M-Mode Ultrasonography

By employing M-mode ultrasonography, it is possible to identify the inspiratory contraction of the muscles, following a similar approach used for assessing the TFdi. Specifically, during the phase of inhalation, an observable augmentation in muscle thickness occurs due to the contraction of muscle fibers. This contraction leads to the upward and forward displacement of the rib cage, while the muscle’s overall mass remains consistent. Parasternal intercostal muscle-thickening fraction (TFic) can be calculated similarly to the diaphragm thickening fraction (TFdi) using the formula below multiplied by 100 [74]:

$$TFic=\frac{\left(Tic-insp\right)-(Tic-exp)}{\left(Tic-exp\right)}$$

#### 2.3.3. Application of Parasternal Intercostal Muscle Ultrasound in the Weaning Process

Numerous studies in the literature have studied the role of using diaphragmatic ultrasound to predict weaning from MV [52,53,54]. Nevertheless, there are few studies that evaluate the use of parasternal intercostal muscle ultrasound to evaluate the weaning process.

As previously noted, patients with diaphragmatic weakness may compensate by exhibiting increased parasternal muscle activity. Dres et al. observed a correlation between TFic values and the inability to successfully complete a SBT in mechanically ventilated patients. Patients who failed the SBT displayed higher TFic values and lower TFdi values compared to those who successfully underwent the trial. Moreover, individuals with diaphragmatic dysfunction exhibited higher TFic values than those without dysfunction, and these values varied based on the degree of ventilation assistance. A TFic measurement exceeding 8% suggested the presence of diaphragm dysfunction, with a measurement surpassing 10% indicating an anticipated failure in the weaning process [70]. In agreement with these findings, Umbrello et al. reported that high TFic values were associated with low TFdi values, thus suggesting the recruitment of intercostal muscles because of increased respiratory workload when there is diaphragmatic dysfunction, as confirmed by the Gilbert index. However, it is significant to note that lower TFdi values may either reflect a low inspiratory effort or an increased inspiratory effort performed by accessory respiratory muscles, based on the level of mechanical support. Using ultrasound to assess TFic could help us distinguish between the two scenarios during weaning from MV. Moreover, this study showed higher TFdi (>30%) and lower TFic (˂5%) in patients without diaphragmatic dysfunction [71]. Umbrello et al. also studied if a change in TFdi or TFic can be used to assess inspiratory effort in critically ill patients when decreasing levels of mechanical support are applied. They found that TFdi provided only an acceptable assessment of inspiratory effort, which significantly improved when patients with diaphragm dysfunction were excluded from the analysis. Moreover, they found that TFic was beneficial in the bedside evaluation of inspiratory effort, particularly in cases where the TFdi was low [71].

Given the preliminary nature of the available evidence, parasternal intercostal ultrasound might be suggested to help clinicians predict weaning outcomes, particularly when combined with other parameters following adequate training and especially in under-assisted patients who use their accessory respiratory muscles. However, evidence is still scarce to establish the best reference values for predicting weaning outcomes with greater confidence. Moreover, we recommend future studies investigate the impact of combining parasternal intercostal ultrasound with diaphragm ultrasound to assess patients’ improvement during the transition from controlled to assisted mode of ventilation and establish evidence-based protocols in the ICU setting.

### 2.4. Ultrasonographic Assessment of the Airway for Weaning Readiness

Patients undergoing endotracheal intubation may sustain various laryngotracheal injuries [76]. One of the most common complications is laryngeal edema, with an incidence ranging from 3 to 30% [77]. Clinically, it can present as an inspiratory stridor or respiratory distress. Sequelae of this complication include reintubation, which, in turn, increases the incidence of nosocomial pneumonia, length of ICU stay, and mortality [78,79].

Multiple studies have suggested possible factors that may raise a patient’s risk for developing laryngeal edema during intubation, such as female sex [80], as well as factors related to the intubation process, including difficult or traumatic intubation, prolonged intubation, and self-extubation [81]. Therefore, an objective evaluation of airway readiness is needed to allow early detection of patients with laryngeal edema and thus decrease the incidence of extubation failure and reintubation.

One such method is the cuff leak test (CLT), which measures air leakage around the endotracheal tube after deflating the cuff, thereby providing an indirect assessment of upper airway patency. This test has been suggested as a simple method for predicting laryngeal edema post-extubation. As observed by Miller and Cole, a reduced cuff leak volume indicates a higher risk of developing post-extubation stridor due to laryngeal edema [82]. However, Engoren et al. found that while the CLT has a high negative predictive value, its positive predictive value is low. Consequently, although the CLT is a safe and straightforward procedure, its controversial results may affect the decision of the physicians, mainly when the CLT yields a positive result [83]. As an alternative, ultrasound has emerged as a valuable and noninvasive tool for assessing and visualizing the upper airways [84]. Laryngeal ultrasound can measure the air column width (ACW), which is represented by the width of the acoustic shadow seen at the level of the vocal cords, both with the endotracheal balloon cuff inflated and deflated, and calculate the air column width difference (ACWD). These measurements have been proposed as a method for predicting post-extubation laryngeal edema.

Ding et al. conducted a prospective study to investigate the correlation between post-extubation stridor and upper airway ultrasound measurements of the ACW. Measurements from 51 patients were obtained within 24 h before the scheduled extubation. The authors reported that patients with an ACW of 4.5 (0.8) mm after cuff deflation went on to develop post-extubation stridor, while patients who did not develop stridor had an ACW of 6.4 (2) mm [85]. Sutherasan et al. performed an observational study and reported that both ACWD and CLT are predictors of the development of laryngeal edema. Furthermore, the researchers determined a cut-off point of 1.6 mm for ACWD, above which extubation may be performed safely. The sensitivity and specificity of this value were 70.6% and 70.2%, respectively. In short, ACWD emerges as a promising tool for anticipating successful extubation in relation to laryngeal edema [86].

In a prospective clinical trial conducted by Sahbal et al., an ultrasound measure of ACWD was compared to CLT for predicting post-extubation laryngeal edema, with stridor serving as a clinical indicator of this condition. Values below 0.9 mm for ACWD were associated with an increased risk of post-extubation stridor. This threshold demonstrated an impressive sensitivity of 88%, specificity of 82%, positive predictive value (PPV) of 86%, and negative predictive value (NPV) of 83%. Additionally, a proposed cut-off point of 110 mL for the CLT was identified, below which the possibility of post-extubation stridor was markedly higher. This 110 mL cut-off exhibited a sensitivity of 68%, a specificity of 89%, a PPV of 69%, and an NPV of 87% [87].

A recent systematic review conducted by Tsai et al., which encompassed 11 observational studies, revealed lower ACWD measurements in patients who developed post-extubation stridor, demonstrating a sensitivity and specificity of 80% and 81%, respectively [88]. These findings underscore the pivotal contribution of ultrasound, particularly ACWD measurement, in predicting post-extubation stridor occurrence following extubation. Nonetheless, it is important to note that additional multicenter studies with standardized definitions and cutoff values are needed to precisely define the role of upper airway ultrasonography in the weaning process.

Lastly, in assessing weaning readiness, we suggest integrating laryngeal ultrasound to assess for laryngeal edema after performing the CLT, particularly when the leak is less than 110 mL. This can aid in determining the necessity of administering corticosteroids to relieve the edema or to proceed with the assessment (see Figure 1).

### 2.5. Hemodynamic Assessment through Echocardiography

Understanding the changes in cardiopulmonary physiology that take place during MV and its discontinuation is essential for promptly identifying weaning failure due to cardiovascular issues and for effectively managing patients. Achieving successful weaning is dependent on the capacity of both the respiratory system and the heart’s pumping function to withstand these changes [89]. Weaning from MV is similar to a cardiac stress test wherein spontaneous ventilation is an exercise for the heart, and so hemodynamic compromise may occur during the weaning process in patients who are critically ill [89].

During the weaning process, there is a shift from positive pressure MV to spontaneous breathing. This shift causes a negative intrathoracic pressure, which increases the venous return pressure gradient, the right ventricular preload, and the left ventricular preload. In addition, the negative pressure raises the left ventricular afterload due to the increased pressure surrounding the left ventricle. Moreover, the shift causes an increase in the work of breathing as well as adrenergic tone, the latter due to increased serum catecholamine levels [90,91]. This in turn may cause cardiovascular dysfunction, which manifests clinically by increasing pulmonary arterial occlusion pressure (PAOP), left ventricular filling pressure, and lastly, pulmonary edema [89,92]. These hemodynamic changes may have damaging consequences in patients suffering from cardiovascular diseases, or they may unmask cardiovascular dysfunction in patients who have normal resting cardiovascular function, affecting the systolic and diastolic functions of the heart during the weaning process [89]. 

Trans-thoracic echocardiography (TTE) is a non-invasive, real-time, and cost-effective means of monitoring key hemodynamic parameters that can be used to predict the outcomes of weaning. TTE may be used to assess the systolic and diastolic function of the heart to predict the outcomes of weaning. The systolic function can be easily assessed by the ejection fraction. Diastolic function assessment can be carried out by (a) analyzing the mitral inflow profile using pulse wave Doppler, where the early wave (E) represents passive ventricular filling and the late wave (A) signifies active atrial contraction, and (b) assessing the mitral annulus through tissue Doppler imaging in individuals without mitral abnormalities, enabling precise evaluation of left ventricular relaxation (e’ wave) and left ventricular filling pressure (E/e’ ratio). Low filling pressures or impaired relaxation patterns are indicated by E/A ≤ 1 and E/e’ ≤ 13. High filling pressures or pseudo-normal patterns are identified when E/A > 1 and E/e’ > 13. The presence of E/A > 2 signifies increased filling pressure with restrictive patterns [13,93]. 

#### 2.5.1. Application of Echocardiography during the Weaning Process

There are numerous studies in the literature discussing the role of hemodynamic parameters in predicting weaning outcomes in mechanically ventilated patients. In a study conducted by Caille et al., researchers aimed to identify predictive indices of weaning failure of cardiac origin. They found out that when examining patients before SBT, patients with an ejection fraction of the left ventricle < 35% and an E/e’ ratio > 7.8 were more likely to experience weaning failure [94]. Additionally, Moschietto et al. concluded that E/e’ may aid in the prediction of weaning failure at the bedside with a cut-off value of 14.5, which had a specificity of 95.8% and a sensitivity of 75%, which suggests an association between diastolic dysfunction and weaning failure. However, in contrast to the findings of Caille et al., they found that systolic dysfunction had no association with weaning outcomes [95]. Moreover, Lamia et al. and Papanikolaou et al. also showed a positive association between TTE parameters of diastolic failure, specifically E/e′, and weaning failure [96,97]. Lately, there have been inconsistent findings reported in the literature. Sanfilippo et al. performed a meta-analysis to assess if there is an association between hemodynamic parameters and the outcomes of weaning from MV. They found that failure of weaning was associated with parameters that indicate worse diastolic function in the left ventricle (E wave, e’ wave, E/e’) and a high E/e’ ratio. No association between left ventricular systolic dysfunction and weaning failure was observed [98]. 

Based on existing literature, assessment of the diastolic function, such as the E wave, e’ wave, and E/e’ ratio, could be essential to assessing a patient’s readiness for ventilator weaning. However, given the paucity of evidence, the literature should focus on clarifying the role of left ventricular ejection fraction and right ventricular function and setting clear E/e’ cut-off values that help us determine patients who are at high risk of weaning failure.

### 2.6. Ultrasonographic Assessment of Respiratory Physiotherapy 

Respiratory physiotherapy stands as an important component in the comprehensive care of critically ill patients within the ICU. The expertise of physiotherapists helps decrease the incidence of complications linked to MV, such as ventilator-associated pneumonia [99], by enhancing lung volumes, removing respiratory secretions, and improving overall ventilation. Some of the most commonly used techniques are percussion, chest vibrations, positioning, mobilization, suctioning, manual hyperinflation, and specialized cough maneuvers [100,101,102]. Nevertheless, to gain the greatest benefit from these techniques, effective assessment tools can be necessary. 

The use of ultrasonography may help identify lung diseases that are responsive to respiratory physiotherapy, as well as monitor the effectiveness of treatment. The tools traditionally used in their assessment are lung auscultation and chest radiographs. However, some studies have shown that the diagnostic accuracy of these tools is limited [103,104]. For that, LUS emerged as an accurate, dependable, and sensitive alternative for diagnosing prevalent chest pathologies and monitoring the effectiveness of respiratory physiotherapy [105,106].

For instance, in cases where ultrasound imaging reveals lung consolidation featuring fluid bronchograms in mechanically ventilated patients, physiotherapists can employ techniques aimed at enhancing expiratory flow rates, such as huffing or exsufflation, in addition to alterations to the ventilator settings as part of their management strategy. Throughout this process, LUS can also be used to assess the efficacy of treatment. Reductions in the size of the consolidation and visualization of air bronchograms instead of fluid bronchograms serve as indicators of the treatment’s effectiveness [105].

In an observational study carried out by Battaglini et al., which focused on assessing the impact of respiratory physiotherapy (RPT) on the oxygenation levels of 20 severely ill COVID-19 patients who were receiving MV during the weaning phase, although RPT led to improved oxygenation in these patients, this improvement was not associated with a statistically significant reduction in LUS scores. This may be attributed to the study’s relatively small sample size and the distinctive pattern of aeration loss commonly observed in COVID-19 patients. Nonetheless, a positive correlation was observed between improved LUS scores and the percentage of lung gas volume as determined by CT scans. This observation suggests that RPT techniques may be particularly advantageous for patients with better lung aeration. These results support the potential role of LUS when assessing lung aeration both before and after RPT [107].

Hansell et al. conducted a prospective cohort study highlighting the advantages of employing LUS in evaluating changes in lung aeration during RPT. They examined 43 mechanically ventilated patients in the ICU and reported that LUS was able to detect lung aeration after respiratory physiotherapy. Nevertheless, it is important to note that this study was not designed to evaluate the efficacy of particular respiratory physiotherapy treatments or to explore the utilization of LUS in guiding physiotherapy practices. Therefore, larger, more precisely designed studies are needed to delineate the exact role of LUS during RPT in mechanically ventilated patients and to establish its reliability [108].

## 3. Conclusions

Ultrasound is a readily accessible, non-invasive imaging modality that can be used in bedside weaning assessments. Although it shows promise, further high-quality research is needed to better establish its role during the weaning process and to set clear cut-off values that can help identify patients who have a high probability of experiencing weaning failure. Additionally, larger, well-designed studies are required to precisely delineate the role of lung ultrasound during respiratory physiotherapy in mechanically ventilated patients and confirm its reliability. 

## Figures and Tables

**Table 1:** Lung ultrasound findings, interpretation, and corresponding scores according to the Bedside Lung Ultrasound in Emergency (BLUE) protocol.

Pattern	Interpretation/Degree of Aeration	Score	
A-lines (horizontal artifacts parallel to the pleural line) or 2 or fewer B-lines.	Normally aerated lung tissue	0	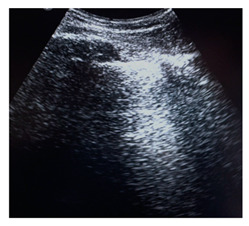
Numerous B-lines (longitudinal artifacts) are either evenly spaced (at intervals of 7 mm) or unevenly spaced or coalescent, but only within a restricted region of the intercostal space.	Moderate decrease in lung tissue aeration	1	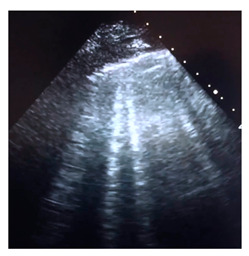
Numerous coalescent B-lines, in widespread areas within the intercostal spaces and noticed in one or multiple intercostal spaces.	Severe decrease in lung tissue aeration	2	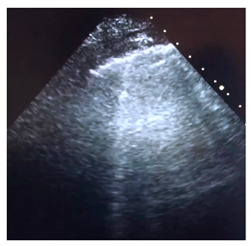
Lung consolidation	Total loss of lung tissue aeration	3	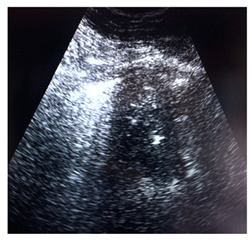

**Table 2:** Differences in definitions of diaphragmatic dysfunction using diaphragm measurements. * indicative of paradoxical diaphragm movement.

	Thickening Fraction (TFdi)	Excursion (E)
Vivier et al. [45]	˂30%	˂10 mm
Lu et al. [46]	˂20%	_____
Kim et al. [47]	_____	˂10 mm or negative *
Mariani et al. [48]	_____	˂11 mm

## Data Availability

Not applicable.

## References

[1] Jaber S., De Jong A. (2023). Weaning from mechanical ventilation in intensive care units: A call for new international consensus guidelines. Lancet Respir. Med..

[2] Girard T.D., Alhazzani W., Kress J.P., Ouellette D.R., Schmidt G.A., Truwit J.D., Burns S.M., Epstein S.K., Esteban A., Fan E. (2017). An official American Thoracic Society/American College of Chest Physicians clinical practice guideline: Liberation from mechanical ventilation in critically ill adults. Rehabilitation protocols, ventilator liberation protocols, and cuff leak tests. Am. J. Respir. Crit. Care Med..

[3] Mayo P., Volpicelli G., Lerolle N., Schreiber A., Doelken P., Vieillard-Baron A. (2016). Ultrasonography evaluation during the weaning process: The heart, the diaphragm, the pleura and the lung. Intensive Care Med..

[4] Beduneau G., Pham T., Schortgen F., Piquilloud L., Zogheib E., Jonas M., Grelon F., Runge I., Terzi N., Grange S. (2017). Epidemiology of weaning outcome according to a new definition. The WIND study. Am. J. Respir. Crit. Care Med..

[5] Heunks L.M., Van Der Hoeven J.G. (2010). Clinical review: The ABC of weaning failure—A structured approach. Crit. Care.

[6] McConville J.F., Kress J.P. (2012). Weaning patients from the ventilator. N. Engl. J. Med..

[7] Matamis D., Soilemezi E., Tsagourias M., Akoumianaki E., Dimassi S., Boroli F., Richard J.-C.M., Brochard L. (2013). Sonographic evaluation of the diaphragm in critically ill patients. Technique and clinical applications. Intensive Care Med..

[8] Bouhemad B., Liu Z.-H., Arbelot C., Zhang M., Ferarri F., Le-Guen M., Girard M., Lu Q., Rouby J.-J. (2010). Ultrasound assessment of antibiotic-induced pulmonary reaeration in ventilator-associated pneumonia. Crit. Care Med..

[9] Baess A.I., Abdallah T.H., Emara D.M., Hassan M. (2016). Diaphragmatic ultrasound as a predictor of successful extubation from mechanical ventilation: Thickness, displacement, or both?. Egypt. J. Bronchol..

[10] Boles J.-M., Bion J., Connors A., Herridge M., Marsh B., Melot C., Pearl R., Silverman H., Stanchina M., Vieillard-Baron A. (2007). Weaning from mechanical ventilation. Eur. Respir. J..

[11] Thille A.W., Gacouin A., Coudroy R., Ehrmann S., Quenot J.-P., Nay M.-A., Guitton C., Contou D., Labro G., Reignier J. (2022). Spontaneous-breathing trials with pressure-support ventilation or a T-piece. N. Engl. J. Med..

[12] Fossé Q., Poulard T., Niérat M.-C., Virolle S., Morawiec E., Hogrel J.-Y., Similowski T., Demoule A., Gennisson J.-L., Bachasson D. (2020). Ultrasound shear wave elastography for assessing diaphragm function in mechanically ventilated patients: A breath-by-breath analysis. Crit. Care.

[13] Santangelo E., Mongodi S., Bouhemad B., Mojoli F. (2022). The weaning from mechanical ventilation: A comprehensive ultrasound approach. Curr. Opin. Crit. Care.

[14] Robba C., Wong A., Poole D., Al Tayar A., Arntfield R.T., Chew M.S., Corradi F., Douflé G., Goffi A., Lamperti M. (2021). Basic ultrasound head-to-toe skills for intensivists in the general and neuro intensive care unit population: Consensus and expert recommendations of the European Society of Intensive Care Medicine. Intensive Care Med..

[15] Bellani G., Mauri T., Pesenti A. (2012). Imaging in acute lung injury and acute respiratory distress syndrome. Curr. Opin. Crit. Care.

[16] Via G., Storti E., Gulati G., Neri L., Mojoli F., Braschi A. (2012). Lung ultrasound in the ICU: From diagnostic instrument to respiratory monitoring tool. Minerva Anestesiol..

[17] Karthika M., Wong D., Nair S.G., Pillai L.V., Mathew C.S. (2019). Lung ultrasound: The emerging role of respiratory therapists. Respir. Care.

[18] Doerschug K.C., Schmidt G.A. (2013). Intensive care ultrasound: III. Lung and pleural ultrasound for the intensivist. Ann. Am. Thorac. Soc..

[19] Mojoli F., Bouhemad B., Mongodi S., Lichtenstein D. (2019). Lung ultrasound for critically ill patients. Am. J. Respir. Crit. Care Med..

[20] Soummer A., Perbet S., Brisson H., Arbelot C., Constantin J.M., Lu Q., Rouby J.J. (2012). Ultrasound assessment of lung aeration loss during a successful weaning trial predicts postextubation distress. Crit. Care Med..

[21] Shoaeir M., Noeam K., Mahrous A., Alaa A. (2016). Lung aeration loss as a predictor of reintubation using lung ultrasound in mechanically ventilated patients. Biolife.

[22] Tenza-Lozano E., Llamas-Alvarez A., Jaimez-Navarro E., Fernández-Sánchez J. (2018). Lung and diaphragm ultrasound as predictors of success in weaning from mechanical ventilation. Crit. Ultrasound J..

[23] Kundu R., Baidya D., Anand R., Maitra S., Soni K., Subramanium R. (2022). Integrated ultrasound protocol in predicting weaning success and extubation failure: A prospective observational study. Anaesthesiol. Intensive Ther..

[24] Tashiro N., Hasegawa T., Nishiwaki H., Ikeda T., Noma H., Levack W., Ota E. (2023). Clinical utility of diaphragmatic ultrasonography for mechanical ventilator weaning in adults: A study protocol for systematic review and meta-analysis. Health Sci. Rep..

[25] Grosu H.B., Im Lee Y., Lee J., Eden E., Eikermann M., Rose K.M. (2012). Diaphragm muscle thinning in patients who are mechanically ventilated. Chest.

[26] Santana P.V., Cardenas L.Z., Albuquerque A.L.P.d. (2023). Diaphragm Ultrasound in Critically Ill Patients on Mechanical Ventilation—Evolving Concepts. Diagnostics.

[27] Doust B.D., Baum J.K., Maklad N.F., Doust V.L. (1975). Ultrasonic evaluation of pleural opacities. Radiology.

[28] Turton P., ALAidarous S., Welters I. (2019). A narrative review of diaphragm ultrasound to predict weaning from mechanical ventilation: Where are we and where are we heading?. Ultrasound J..

[29] Haaksma M., Atmowihardjo L., Heunks L., Spoelstra-de Man A., Tuinman P. (2018). Ultrasound imaging of the diaphragm: Facts and future. A guide for the bedside clinician. Neth. J. Crit. Care.

[30] De Rosa S., Umbrello M., Pelosi P., Battaglini D. (2023). Update on Lean Body Mass Diagnostic Assessment in Critical Illness. Diagnostics.

[31] Haaksma M.E., Smit J.M., Boussuges A., Demoule A., Dres M., Ferrari G., Formenti P., Goligher E.C., Heunks L., Lim E.H. (2022). EXpert consensus On Diaphragm UltraSonography in the critically ill (EXODUS): A Delphi consensus statement on the measurement of diaphragm ultrasound-derived parameters in a critical care setting. Crit. Care.

[32] Tuinman P.R., Jonkman A.H., Dres M., Shi Z.-H., Goligher E.C., Goffi A., de Korte C., Demoule A., Heunks L. (2020). Respiratory muscle ultrasonography: Methodology, basic and advanced principles and clinical applications in ICU and ED patients—A narrative review. Intensive Care Med..

[33] Gottesman E., McCool F.D. (1997). Ultrasound evaluation of the paralyzed diaphragm. Am. J. Respir. Crit. Care Med..

[34] Cohn D., Benditt J.O., Eveloff S., McCool F.D. (1997). Diaphragm thickening during inspiration. J. Appl. Physiol..

[35] Boussuges A., Rives S., Finance J., Brégeon F. (2020). Assessment of diaphragmatic function by ultrasonography: Current approach and perspectives. World J. Clin. Cases.

[36] Goligher E.C., Laghi F., Detsky M.E., Farias P., Murray A., Brace D., Brochard L.J., Sebastien-Bolz S., Rubenfeld G.D., Kavanagh B.P. (2015). Measuring diaphragm thickness with ultrasound in mechanically ventilated patients: Feasibility, reproducibility and validity. Intensive Care Med..

[37] Schepens T., Verbrugghe W., Dams K., Corthouts B., Parizel P.M., Jorens P.G. (2015). The course of diaphragm atrophy in ventilated patients assessed with ultrasound: A longitudinal cohort study. Crit. Care.

[38] Santana P.V., Cardenas L.Z., Albuquerque A.L.P.d., Carvalho C.R.R.d., Caruso P. (2020). Diaphragmatic ultrasound: A review of its methodological aspects and clinical uses. J. Bras. Pneumol..

[39] Haji K., Royse A., Green C., Botha J., Canty D., Royse C. (2016). Interpreting diaphragmatic movement with bedside imaging, review article. J. Crit. Care.

[40] Houston J.G., Morris A.D., Howie C.A., Reid J.L., McMillan N. (1992). Technical report: Quantitative assessment of diaphragmatic movement—A reproducible method using ultrasound. Clin. Radiol..

[41] Haji K., Royse A., Tharmaraj D., Haji D., Botha J., Royse C. (2015). Diaphragmatic regional displacement assessed by ultrasound and correlated to subphrenic organ movement in the critically ill patients—An observational study. J. Crit. Care.

[42] Toledo N.S., Kodaira S.K., Massarollo P.C., Pereira O.I., Dalmas J.C., Cerri G.G., Buchpiguel C.A. (2006). Left hemidiaphragmatic mobility: Assessment with ultrasonographic measurement of the craniocaudal displacement of the splenic hilum and the inferior pole of the spleen. J. Ultrasound Med..

[43] Scarlata S., Mancini D., Laudisio A., Benigni A., Raffaele A.I. (2018). Reproducibility and Clinical Correlates of Supine Diaphragmatic Motion Measured by M-Mode Ultrasonography in Healthy Volunteers. Respiration.

[44] Boussuges A., Gole Y., Blanc P. (2009). Diaphragmatic motion studied by m-mode ultrasonography: Methods, reproducibility, and normal values. Chest.

[45] Vivier E., Muller M., Putegnat J.-B., Steyer J., Barrau S., Boissier F., Bourdin G., Mekontso-Dessap A., Levrat A., Pommier C. (2019). Inability of diaphragm ultrasound to predict extubation failure: A multicenter study. Chest.

[46] Lu Z., Xu Q., Yuan Y., Zhang G., Guo F., Ge H. (2016). Diaphragmatic dysfunction is characterized by increased duration of mechanical ventilation in subjects with prolonged weaning. Respir. Care.

[47] Kim W.Y., Suh H.J., Hong S.-B., Koh Y., Lim C.-M. (2011). Diaphragm dysfunction assessed by ultrasonography: Influence on weaning from mechanical ventilation. Crit. Care Med..

[48] Mariani L.F., Bedel J., Gros A., Lerolle N., Milojevic K., Laurent V., Hilly J., Troché G., Bedos J.P., Planquette B. (2016). Ultrasonography for screening and follow-up of diaphragmatic dysfunction in the ICU: A pilot study. J. Intensive Care Med..

[49] Alam M.J., Roy S., Iktidar M.A., Padma F.K., Nipun K.I., Chowdhury S., Nath R.K., Rashid H.-O. (2022). Diaphragm ultrasound as a better predictor of successful extubation from mechanical ventilation than rapid shallow breathing index. Acute Crit. Care.

[50] Eltrabili H.H., Hasanin A.M., Soliman M.S., Lotfy A.M., Hamimy W.I., Mukhtar A.M. (2019). Evaluation of diaphragmatic ultrasound indices as predictors of successful liberation from mechanical ventilation in subjects with abdominal sepsis. Respir. Care.

[51] Elshazly M.I., Elkorashy R.I., Ismail M.S., Ismail J.H., Assal H.H. (2020). Role of bedside ultrasonography in assessment of diaphragm function as a predictor of success of weaning in mechanically ventilated patients. Tuberc. Respir. Dis..

[52] Li S., Chen Z., Yan W. (2021). Application of bedside ultrasound in predicting the outcome of weaning from mechanical ventilation in elderly patients. BMC Pulm. Med..

[53] Gok F., Mercan A., Kilicaslan A., Sarkilar G., Yosunkaya A., Funda G., Kılıçaslan A. (2021). Diaphragm and lung ultrasonography during weaning from mechanical ventilation in critically ill patients. Cureus.

[54] Blumhof S., Wheeler D., Thomas K., McCool F.D., Mora J. (2016). Change in diaphragmatic thickness during the respiratory cycle predicts extubation success at various levels of pressure support ventilation. Lung.

[55] Le Neindre A., Philippart F., Luperto M., Wormser J., Morel-Sapene J., Aho S.L., Mongodi S., Mojoli F., Bouhemad B. (2021). Diagnostic accuracy of diaphragm ultrasound to predict weaning outcome: A systematic review and meta-analysis. Int. J. Nurs. Stud..

[56] Mahmoodpoor A., Fouladi S., Ramouz A., Shadvar K., Ostadi Z., Soleimanpour H. (2022). Diaphragm ultrasound to predict weaning outcome: Systematic review and meta-analysis. Anaesthesiol. Intensive Ther..

[57] Llamas-Alvarez A.M., Tenza-Lozano E.M., Latour-Perez J. (2017). Diaphragm and lung ultrasound to predict weaning outcome: Systematic review and meta-analysis. Chest.

[58] Parada-Gereda H.M., Tibaduiza A.L., Rico-Mendoza A., Molano-Franco D., Nieto V.H., Arias-Ortiz W.A., Perez-Terán P., Masclans J.R. (2023). Effectiveness of diaphragmatic ultrasound as a predictor of successful weaning from mechanical ventilation: A systematic review and meta-analysis. Crit. Care.

[59] Esteban A., Anzueto A., Frutos F., Alía I., Brochard L., Stewart T.E., Benito S., Epstein S.K., Apezteguía C., Nightingale P. (2002). Characteristics and outcomes in adult patients receiving mechanical ventilation: A 28-day international study. JAMA.

[60] Hess D.R. (2005). Ventilator waveforms and the physiology of pressure support ventilation. Respir. Care.

[61] Thille A.W., Cabello B., Galia F., Lyazidi A., Brochard L. (2008). Reduction of patient-ventilator asynchrony by reducing tidal volume during pressure-support ventilation. Intensive Care Med..

[62] Hudson M.B., Smuder A.J., Nelson W.B., Bruells C.S., Levine S., Powers S.K. (2012). Both high level pressure support ventilation and controlled mechanical ventilation induce diaphragm dysfunction and atrophy. Crit. Care Med..

[63] Umbrello M., Formenti P., Longhi D., Galimberti A., Piva I., Pezzi A., Mistraletti G., Marini J.J., Iapichino G. (2015). Diaphragm ultrasound as indicator of respiratory effort in critically ill patients undergoing assisted mechanical ventilation: A pilot clinical study. Crit. Care.

[64] Cammarota G., Boniolo E., Tarquini R., Vaschetto R. (2020). Diaphragmatic excursion tissue Doppler sonographic assessment. Intensive Care Med..

[65] Maurizio R., Rinaldi V.E., Camerini P.G., Salvatori C., Leonardi A., Bini V. (2019). Right Diaphragmatic Peak Motion Velocities on Pulsed Wave Tissue Doppler Imaging in Neonates: Method, Reproducibility, and Reference Values. J. Ultrasound Med..

[66] Soilemezi E., Savvidou S., Sotiriou P., Smyrniotis D., Tsagourias M., Matamis D. (2020). Tissue Doppler imaging of the diaphragm in healthy subjects and critically ill patients. Am. J. Respir. Crit. Care Med..

[67] Cammarota G., Boniolo E., Santangelo E., De Vita N., Verdina F., Crudo S., Sguazzotti I., Perucca R., Messina A., Zanoni M. (2021). Diaphragmatic kinetics assessment by tissue doppler imaging and extubation outcome. Respir. Care.

[68] Oppersma E., Hatam N., Doorduin J., van der Hoeven J.G., Marx G., Goetzenich A., Fritsch S., Heunks L.M.A., Bruells C.S. (2017). Functional assessment of the diaphragm by speckle tracking ultrasound during inspiratory loading. J. Appl. Physiol..

[69] Bachasson D., Dres M., Niérat M.-C., Gennisson J.-L., Hogrel J.-Y., Doorduin J., Similowski T. (2019). Diaphragm shear modulus reflects transdiaphragmatic pressure during isovolumetric inspiratory efforts and ventilation against inspiratory loading. J. Appl. Physiol..

[70] Dres M., Dubé B.-P., Goligher E., Vorona S., Demiri S., Morawiec E., Mayaux J., Brochard L., Similowski T., Demoule A. (2020). Usefulness of parasternal intercostal muscle ultrasound during weaning from mechanical ventilation. Anesthesiology.

[71] Umbrello M., Formenti P., Lusardi A.C., Guanziroli M., Caccioppola A., Coppola S., Chiumello D. (2020). Oesophageal pressure and respiratory muscle ultrasonographic measurements indicate inspiratory effort during pressure support ventilation. Br. J. Anaesth..

[72] Lichtenstein D.A. (2014). Lung ultrasound in the critically ill. Ann. Intensive Care.

[73] Wallbridge P., Parry S.M., Das S., Law C., Hammerschlag G., Irving L., Hew M., Steinfort D. (2018). Parasternal intercostal muscle ultrasound in chronic obstructive pulmonary disease correlates with spirometric severity. Sci. Rep..

[74] Formenti P., Umbrello M., Dres M., Chiumello D. (2020). Ultrasonographic assessment of parasternal intercostal muscles during mechanical ventilation. Ann. Intensive Care.

[75] Sarwal A., Parry S.M., Berry M.J., Hsu F.-C., Lewis M.T., Justus N.W., Morris P.E., Denehy L., Berney S., Dhar S. (2015). Interobserver reliability of quantitative muscle sonographic analysis in the critically ill population. J. Ultrasound Med..

[76] Thomas R., Kumar E.V., Kameswaran M., Shamim A., Al Ghamdii S., Mummigatty A.P., Okafor B. (1995). Post intubation laryngeal sequelae in an intensive care unit. J. Laryngol. Otol..

[77] Fan T., Wang G., Mao B., Xiong Z., Zhang Y., Liu X., Wang L., Yang S. (2008). Prophylactic administration of parenteral steroids for preventing airway complications after extubation in adults: Meta-analysis of randomised placebo controlled trials. BMJ.

[78] Epstein S.K., Ciubotaru R.L. (1998). Independent effects of etiology of failure and time to reintubation on outcome for patients failing extubation. Am. J. Respir. Crit. Care Med..

[79] Torres A., Gatell J.M., Aznar E., El-Ebiary M., Puig de la Bellacasa J., González J., Ferrer M., Rodriguez-Roisin R. (1995). Re-intubation increases the risk of nosocomial pneumonia in patients needing mechanical ventilation. Am. J. Respir. Crit. Care Med..

[80] Ho L., Harn H., Lien T., Hu P., Wang J. (1996). Postextubation laryngeal edema in adults risk factor evaluation and prevention by hydrocortisone. Intensive Care Med..

[81] Jaber S., Chanques G., Matecki S., Ramonatxo M., Vergne C., Souche B., Perrigault P.-F., Eledjam J.-J. (2003). Post-extubation stridor in intensive care unit patients: Risk factors evaluation and importance of the cuff-leak test. Intensive Care Med..

[82] Miller R.L., Cole R.P. (1996). Association between reduced cuff leak volume and postextubation stridor. Chest.

[83] Engoren M. (1999). Evaluation of the cuff-leak test in a cardiac surgery population. Chest.

[84] Shih J.-Y., Lee L.-N., Wu H.-D., Yu C.-J., Wang H.-C., Chang Y.-L., Yang P.-C. (1997). Sonographic imaging of the trachea. J. Ultrasound Med..

[85] Ding L., Wang H., Wu H.-D., Chang C., Yang P. (2006). Laryngeal ultrasound: A useful method in predicting post-extubation stridor. A pilot study. Eur. Respir. J..

[86] Sutherasan Y., Theerawit P., Hongphanut T., Kiatboonsri C., Kiatboonsri S. (2013). Predicting laryngeal edema in intubated patients by portable intensive care unit ultrasound. J. Crit. Care.

[87] Sahbal M.A., Mohamed K.A., Zaghla H.H., Kenawy M.M. (2017). Laryngeal ultrasound versus cuff leak test in prediction of post-extubation stridor. Egypt. J. Crit. Care Med..

[88] Tsai W.-W., Hung K.-C., Huang Y.-T., Yu C.-H., Lin C.-H., Chen I.-W., Sun C.-K. (2023). Diagnostic efficacy of sonographic measurement of laryngeal air column width difference for predicting the risk of post-extubation stridor: A meta-analysis of observational studies. Front. Med..

[89] Routsi C., Stanopoulos I., Kokkoris S., Sideris A., Zakynthinos S. (2019). Weaning failure of cardiovascular origin: How to suspect, detect and treat—A review of the literature. Ann. Intensive Care.

[90] Oh T., Bhatt S., Lin E., Hutchinson R., Low J. (1991). Plasma catecholamines and oxygen consumption during weaning from mechanical ventilation. Intensive Care Med..

[91] Lemaire F., Teboul J.-L., Cinotti L., Giotto G., Abrouk F., Steg G., Macquin-Mavier I., Zapol W.M. (1988). Acute left ventricular dysfunction during unsuccessful weaning from mechanical ventilation. Anesthesiology.

[92] Teboul J.-L. (2014). Weaning-induced cardiac dysfunction: Where are we today?. Intensive Care Med..

[93] Nagueh S.F., Smiseth O.A., Appleton C.P., Byrd B.F., Dokainish H., Edvardsen T., Flachskampf F.A., Gillebert T.C., Klein A.L., Lancellotti P. (2016). Recommendations for the evaluation of left ventricular diastolic function by echocardiography: An update from the American Society of Echocardiography and the European Association of Cardiovascular Imaging. Eur. J. Echocardiogr..

[94] Caille V., Amiel J.-B., Charron C., Belliard G., Vieillard-Baron A., Vignon P. (2010). Echocardiography: A help in the weaning process. Crit. Care.

[95] Moschietto S., Doyen D., Grech L., Dellamonica J., Hyvernat H., Bernardin G. (2012). Transthoracic echocardiography with Doppler tissue imaging predicts weaning failure from mechanical ventilation: Evolution of the left ventricle relaxation rate during a spontaneous breathing trial is the key factor in weaning outcome. Crit. Care.

[96] Lamia B., Maizel J., Ochagavia A., Chemla D., Osman D., Richard C., Teboul J.-L. (2009). Echocardiographic diagnosis of pulmonary artery occlusion pressure elevation during weaning from mechanical ventilation. Crit. Care Med..

[97] Papanikolaou J., Makris D., Saranteas T., Karakitsos D., Zintzaras E., Karabinis A., Kostopanagiotou G., Zakynthinos E. (2011). New insights into weaning from mechanical ventilation: Left ventricular diastolic dysfunction is a key player. Intensive Care Med..

[98] Sanfilippo F., Di Falco D., Noto A., Santonocito C., Morelli A., Bignami E., Scolletta S., Vieillard-Baron A., Astuto M. (2021). Association of weaning failure from mechanical ventilation with transthoracic echocardiography parameters: A systematic review and meta-analysis. Br. J. Anaesth..

[99] Ntoumenopoulos G., Presneill J., McElholum M., Cade J. (2002). Chest physiotherapy for the prevention of ventilator-associated pneumonia. Intensive Care Med..

[100] Choi J.S.-P., Jones A.Y.-M. (2005). Effects of manual hyperinflation and suctioning on respiratory mechanics in mechanically ventilated patients with ventilator-associated pneumonia. Aust. J. Physiother..

[101] Raoof S., Chowdhrey N., Raoof S., Feuerman M., King A., Sriraman R., Khan F.A. (1999). Effect of combined kinetic therapy and percussion therapy on the resolution of atelectasis in critically ill patients. Chest.

[102] Pattanshetty R., Gaude G. (2011). Effect of multimodality chest physiotherapy on the rate of recovery and prevention of complications in patients with mechanical ventilation: A prospective study in medical and surgical intensive care units. Indian. J. Med. Sci..

[103] Leech M., Bissett B., Kot M., Ntoumenopoulos G. (2015). Lung ultrasound for critical care physiotherapists: A narrative review. Physiother. Res. Int..

[104] Lichtenstein D., Goldstein I., Mourgeon E., Cluzel P., Grenier P., Rouby J.-J. (2004). Comparative diagnostic performances of auscultation, chest radiography, and lung ultrasonography in acute respiratory distress syndrome. J. Am. Soc. Anesthesiol..

[105] Le Neindre A., Mongodi S., Philippart F., Bouhemad B. (2016). Thoracic ultrasound: Potential new tool for physiotherapists in respiratory management. A narrative review. J. Crit. Care.

[106] Mohsen A., Samy W.A., El-Azizy H.M., Shehata M.H. (2018). Lung ultrasound in intensive care unit: A prospective comparative study with bedside chest radiography using computed tomography of chest as a gold standard. Res. Opin. Anesth. Intensive Care.

[107] Battaglini D., Caiffa S., Gasti G., Ciaravolo E., Robba C., Herrmann J., Gerard S.E., Bassetti M., Pelosi P., Ball L. (2021). An Experimental Pre-Post Study on the Efficacy of Respiratory Physiotherapy in Severe Critically III COVID-19 Patients. J. Clin. Med..

[108] Hansell L., Milross M., Delaney A., Koo C.M., Tian D.H., Ntoumenopoulos G. (2023). Quantification of changes in lung aeration associated with physiotherapy using lung ultrasound in mechanically ventilated patients: A prospective cohort study. Physiotherapy.

